# Atypical Fentanyl Transdermal Patch Consumption and Fatalities: Case Report and Literature Review

**DOI:** 10.3390/toxics11010046

**Published:** 2022-12-31

**Authors:** Federico Manetti, Maria Chiara David, Sara Gariglio, Francesca Consalvo, Martina Padovano, Matteo Scopetti, Antonio Grande, Alessandro Santurro

**Affiliations:** 1 Department of Anatomical, Histological, Forensic and Orthopedic Sciences, Sapienza University of Rome, 00185 Rome, Italy; 2 Ministry of the Interior, Department of Public Security, Health Central Directorate, State Police, 00185 Rome, Italy; 3 DIFAR—Department of Pharmacy, University of Genova, 16148 Genova, Italy; 4 Department of Medical Surgical Sciences and Translational Medicine, Sapienza University of Rome, 00189 Rome, Italy; 5 Department of Public Security, Anti-Crime Central Directorate, Scientific Police Service, 00174 Rome, Italy; 6 Department of Medicine, Surgery and Dentistry, University of Salerno, 84081 Baronissi, Italy

**Keywords:** fentanyl, morphine, transdermal patch, protective film, autopsy, forensic toxicology, forensic pathology

## Abstract

Fentanyl is a synthetic L-opioid receptor agonist, approximately 100 times more potent than morphine, that is experiencing an upward trend in the field of abuse. Fentanyl patches’ abusive consumption can occur either by transdermal absorption or through other atypical and ingenious routes. In the present case, a 29-year-old man with a history of illicit drug use was found dead in a suburban neighborhood of an Italian city. At autopsy, lungs appeared increased in weight and showed minute subpleural hemorrhages. Airways contained abundant reddish foamy material; in addition, a fentanyl patch protective film was found inside the left main bronchus. Toxicological analysis revealed the presence of morphine, fentanyl, BEG and ethyl alcohol in peripheric blood; 6-MAM was also revealed in urine. Findings collected during post-mortem investigations allowed us to identify fentanyl consumption as the cause of death. Fentanyl consumption presumably took place by chewing of a transdermal patch, with subsequent aspiration of the protective film. The pathophysiology of death can be identified as combined respiratory failure—both central suppression and a fentanyl-induced increase in muscular stiffness; a further minor contribution may be identified in the mechanical airflow obstruction caused by the presence of the protective film at the bronchial level.

## 1. Introduction

Fentanyl is a synthetic L-opioid receptor agonist, approximately 100 times more potent than morphine per dose [1,2], widely used as a narcotic supplement in anesthesia and in the management of acute and severe chronic pain [3]. However, by virtue of its effectiveness and diffusion, fentanyl is experiencing an important upward trend also in the field of abuse, to the point of constituting, alongside the well-known classical opioids, a current and concrete problem of public order in many countries [4,5,6,7,8,9,10,11,12].

The consumption of fentanyl for recreational use includes its addition to other illicit drugs (such as heroin and cocaine) in order to enhance their effect at a low cost, as well as the use of formulations for medical purposes available on the market [3]. As regards the latter aspect, a characteristic and recurrent feature detectable in the international forensic toxicological literature is the widespread use of fentanyl transdermal patches, not only as a prescription in home therapies for the control of chronic pain, but also for recreational consumption.

In the presented case, the orientation towards a diagnosis of death due to the intake of a fentanyl transdermal patch by an atypical route of administration was provided by the typical signs of acute respiratory failure referable to exogenous intoxication, and the finding of a patch protective film inside the left main bronchus; subsequent histological and toxicological investigations, corroborated by available scientific evidence, allowed us to confirm the hypothesis proposed at the autoptic table.

## 2. Case Report

A 29-year-old man with a history of illicit drug use was found dead in a suburban neighborhood of an Italian major city. At the time of scene investigation, the corpse was laid on a wooden table, along a sidewalk, in lateral decubitus. Early-stage postmortem lividity was expressed consistently with the position assumed by the body. Clear residues of vomit, not observed elsewhere on the scene, were detected on the face and the clothes; whitish foamy material was found in proximity to nasal orifices. At the clothes’ examination, a pack of alprazolam and four doses of hashish were found in the right pocket of the sweatshirt.

Preliminary judicial investigations made it possible to suspect that the man was abandoned along the sidewalk after he lost consciousness, while attending a party in which illicit drug consumption had taken place. Judicial autopsy was disposed by the prosecutor and performed approximately 48 h after the discovery of the corpse, in order to clarify the causes of death and exclude criminal conduct.

On external examination, his body was 173 cm in height and 80 kg in weight. Any type of lesion attributable to violent causes was excluded during the inspection.

At autopsy, brain weight was within normal limits (1325 g) and showed on its surface, besides congestion of leptomeningeal vessels, flattening of convolutions and superficialization of sulci, suggestive of cerebral edema. Trachea contained reddish foamy material and minute blood clots.

**Figure 1:**
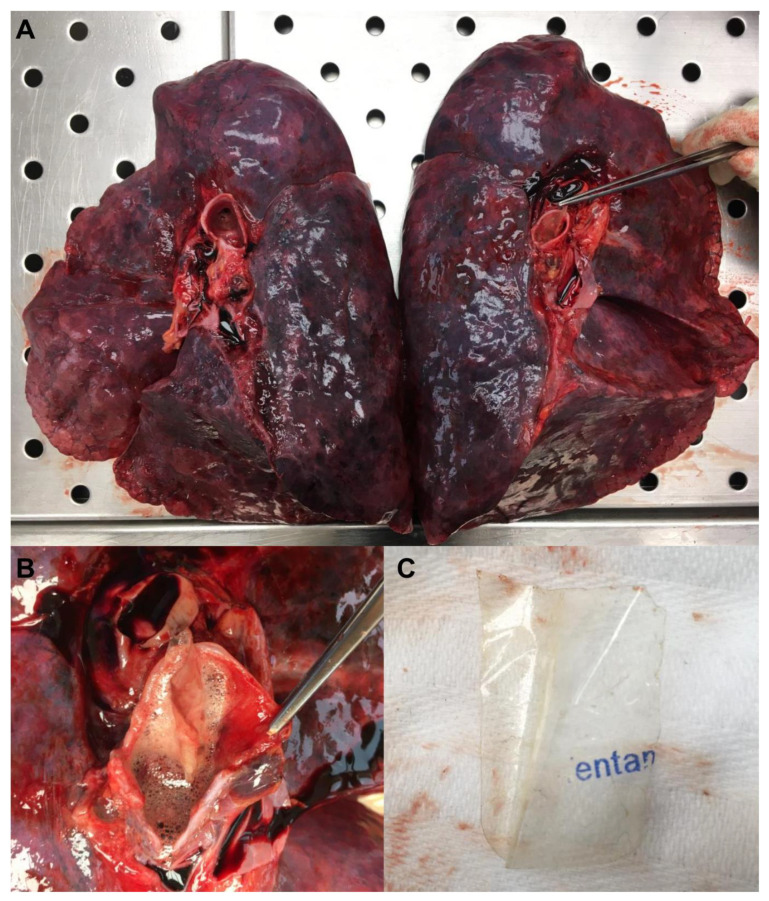
Autopsy Findings: (**A**) Macroscopic aspect of lungs. (**B**) Detail of left bronchus with fentanyl patch protective film. (**C**) Fentanyl patch protective film.

Lungs appeared increased in weight (848 g and 827 g, respectively, left and right), were regular in size and shape, and showed minute subpleural hemorrhages (Figure 1A). The opening of the airways allowed us to detect abundant reddish foamy material; in addition, a plastic protective film with the inscription “entan” was found inside the left main bronchus (Figure 1B,C). Edema and vascular congestion were revealed at the examination of the parenchyma.

The stomach contained 50 mL of undifferentiated food material; it was not possible to detect the presence of pills or other findings attributable to drugs.

Examination of other organs revealed non-specific signs of asphyxia and marked vascular congestion, in the absence of further noteworthy or pathological findings. Immunochemical drug screening for urine samples was positive for cocaine, morphine, methadone, cannabinoids (THC), and benzodiazepines.

During the investigations, biological samples were taken for histopathological and quantitative toxicological tests. Microscopic investigation of formalin-fixed paraffin-embedded (FFPE) tissue specimens after section and hematoxylin and eosin (H&E) staining allowed us to confirm the generalized vascular congestion macroscopically detected. Brain samples showed both intracellular and extracellular cerebral edema. Heart specimens were characterized by a moderate increase in perivascular and interstitial fibrous connective tissue representation, histologically relevant in consideration of the young age of the man. Lungs were characterized by the presence of abundant amorphous eosinophilic material in alveoli, indicative of alveolar edema, sometimes associated with erythrocyte alveolar infiltrations; in some fields, spots of acute emphysema were also noticed. No further findings of interest were revealed at the examination of the remaining tissue samples. In light of the aforementioned findings, the death was therefore attributed to acute respiratory failure referable to exogenous intoxication.

## 3. Materials and Methods

The toxicological investigations carried out concerned the search for the most common substances of forensic toxicological interest, and were conducted in accordance with the indications of the Group of Italian Forensic Toxicologists (GIFT) and the scientific evidence inferable from the international literature [13,14,15,16,17,18,19,20,21].

### 3.1. Chemicals and Reagents

Methanol solutions of fentanyl, methadone, EDDP, morphine, 6-monoacetylmorphine (6-MAM), cocaine, benzoilecgonine, and alprazolam and internal standard solutions of fentanyl-d5, methadone-d9, EDDP-d3, morphine-d3, 6-MAM-d3, cocaine-d3, benzoilecgonine-d3, and alprazolam-d4 (0.1 mg/mL), were purchased from Lipomed AG (Arlesheim, Switzerland). All solutions were stored in the refrigerator at +2–+4 °C when not in use. Mixed working standard solutions were prepared by combining the standards and aliquots of each primary solution and diluting them with methanol. Methanol, dichlormethane, isopropanol, ammonium hydroxide, and others were purchased from Carlo Erba Reagents (Val-de-Reuil, France). Extraction Columns Clean Screen XCEL I and Selectra-SIL BSTFA w/1%TMCS were purchased from UCT (Bristol, PA, USA).

### 3.2. Calibration Curves

All calibration curves were prepared by spiking blank blood with appropriate volumes of standard and internal standard solutions. The amount of internal standard used was the same for the calibration curve and the real samples. Calibration curves were prepared on 0.5 mL of matrix; appropriate dilutions were applied if needed for the analysis of real samples.

#### 3.2.1. Blood Alcohol

Calibration curve points were 0.25, 0.5, 1, 2, 4 g/L.

#### 3.2.2. Fentanyl

Calibration curve points were 2.5, 5, 10, 50, 100 ng/mL.

### 3.3. Sample Pretreatment

#### 3.3.1. Fentanyl, Cocaine, Benzoylecgonine, Morphine, 6-Monoacetylmorphine

Samples were extracted using the extraction column Screen XCEL I (UCT). The extraction procedure was as follows: an appropriate volume of sample was diluted with phosphate buffer (pH 6) and added directly to the column without any preconditioning, allowed to flow by gravity, dried for 1 min, washed with 1 mL of 2% glacial acetic acid/98% methanol, dried for 5 min, and eluted with dichloromethane/isopropanol/ammonium hydroxide (78/20/2). The eluate was then evaporated to dryness under a gentle stream of N_2_ gas at 40 °C, and the residue was derivatized with 50 µL of BSTFA 1% TMCS 70 °C per 20 min. Finally, 1 µL of the solution was injected into the GC-MS.

#### 3.3.2. Alprazolam

Samples were extracted using the extraction column Screen XCEL I (UCT). The extraction procedure was as follows: an appropriate volume of sample was diluted with phosphate buffer (pH 6) and added directly to the column without any preconditioning, allowed to flow by gravity, dried for 1 min, washed with 1 mL of dichloromethane, dried for 5 min, and eluted with ethyl acetate:ammonia (98:2). The eluate was then evaporated to dryness under a gentle stream of N_2_ gas at 40 °C, and the residue was derivatized with 50 µL of BSTFA 1% TMCS 70 °C per 20 min. Finally, 1 µL of the solution was injected into the GC-MS.

#### 3.3.3. Methadone, EDDP

An appropriate volume of sample was diluted with 0.5 mL deionized water and combined with 50 µL of 10 M ammonium hydroxide aqueous solution and an appropriate amount of internal standard. After vortexing for 30 mins 3 mL of ethyl acetate was added as an extraction solvent. Liquid–liquid extraction (LLE) was carried out by stirring the sample for 15 min on an automatic vortex and then centrifuging it at 3000 RPM for 5 min. The organic phase was recovered, transferred into a disposable vial, and evaporated to dryness with a flow of N_2_. The samples were reconstituted using 50 µL of ethyl acetate. Finally, 1 µL of the solution was injected into the GC-MS.

#### 3.3.4. Blood Alcohol

First, 0.5 mL of sample was added to a glass vial for head space analysis and mixed with 0.5 mL of a 1 g/L solution of isopropylic alcohol as an internal standard. The sample was then closed with a tight stopper and mixed.

### 3.4. GC-MS Conditions

All analyses were performed with a GC system, the Agilent 7820 A, coupled with a single quadrupole mass spectrometer, MSD-5975. Chromatographic separation was conducted with an Agilent GC Column HP-5MS (0.25 µm, 0.2 mm i.d., 20 m) (Agilent Technologies, Santa Clara, CA, USA).

#### 3.4.1. Fentanyl

The injection temperature was 280 °C and the injection volume was 1 µL. The injection mode was split 15:1. The oven was programmed from 160 °C for 1 min, ramped at 20 °C/min to 290 °C, and held for 10 min. Fentanyl retention time was 8.02; Select Ion Monitoring (SIM) values were m/z 189, 194, 146, 151 (qualifiers), and m/z 245 (quantifier); fentanyl-d5 SIM was m/z 250 (quantifier).

#### 3.4.2. Cocaine, Benzoylecgonine, Morphine, 6-Monoacethylmorphine, Methadone, EDDP

The injection temperature was 280 °C and the injection volume was 1 µL. The injection mode was split 15:1. The oven was programmed from 140 °C for 1 min, ramped at 20 °C/min to 290 °C, and held for 6 min. EDDP retention time was 5.90 min; its SIM values were m/z 277, 262 (qualifiers), and m/z 276 (quantifier). Methadone retention time was 6.42 min; its SIM values were m/z 223, 294 (qualifiers), and m/z 72 (quantifier). Methadone-d9 SIM was m/z 78 (quantifier). Cocaine retention time was 6.70 min; its SIM values were m/z 272, 82 (qualifiers) and m/z 303 (quantifier). Cocaine-d3 SIM was m/z 306 (quantifier). Benzoylecgonine retention time was 7.00 min; its SIM values were m/z 240, 82 (qualifiers), and m/z 361 (quantifier). Bezoylecgonine-d3 SIM value was m/z 364 (quantifier). Morphine retention time was 7.99 min; its SIM values were m/z 236, 401 (qualifiers), and m/z 429 (quantifier); morphine-d3 was m/z 433 (quantifier). 6-Monoacetylmorphine retention time was 8.25 min; its SIM values were m/z 340, 287 (qualifiers), and m/z 399 (quantifier); morphine-d3 was m/z 402 (quantifier).

#### 3.4.3. Alprazolam

The injection temperature was 260 °C and the injection volume was 1 µL. The injection mode was split 15:1. The oven was programmed from 120 °C for 1 min, ramped at 15 °C/min to 290 °C, and held for 5 min. Alprazolam retention time was 13.41 min; its SIM values were m/z 279, 204 (qualifiers), and m/z 308 (quantifier). Alprazolam-d5 SIM was m/z 313 (quantifier).

### 3.5. HS-GC-FID Conditions

Analyses were performed with a GC system, the Agilent 7820A, coupled with a HS Agilent 7694. Chromatographic separation was performed with an Agilent GC Column HP-B ALC (0.32 µm, 0.2 mm i.d., 7.5 mt) (Agilent Technologies, Santa Clara, CA, USA). The manifold temperature was 75 °C, the head-space (HS) temperature was 70 °C, and the oven was programmed at 85 °C and held for 5 min, which was also the total duration of analysis. Ethanol retention time was 0.76 min; IS retention time was 1.26 min.

### 3.6. Method Validation

The method for fentanyl analysis was validated for this study according to the guidelines of the Group of Italian Forensic Toxicologists (GIFT) [22]. Calibration curves revealed good linearity (R^2^ > 0.997). The recovery ranged from 80 to 89%. Fentanyl’s lower limit of quantification (LLOQ) was 2.5 ng/mL, while the limit of detection (LOD) was 1.5 ng/mL. The LOD was defined as the lowest concentration giving a response at least three times higher than the average of the baseline noise, while the LLOQ was defined as the lowest concentration that could be measured with an intra-assay precision CV% and relative bias less than 20%. The LLOQ also matches the last level of the calibration curve. The results obtained with this method using whole blood were linear and sensitive; the accuracy and precision of validation data were within +/− 15%.

All other methods were already in use in the laboratory of this group prior to this study. All LODs and LOQs can be found in Table 1.

## 4. Results

The results revealed the presence of ethyl alcohol, methadone, 2-ethylidine-1,5-dimethyl-3,3-diphenylpyrrolidine (EDDP), morphine, 6-monoacetylmorphine (6-MAM), cocaine, benzoylecgonine (BEG), fentanyl, and alprazolam, as reported in Table 2.

## 5. Discussion

Fentanyl patches are available in different dosages and formulations. “Membrane-controlled” patches consist of a reservoir (hence the name of “reservoir design”) containing the active ingredient in gel form in a formulation containing ethanol USP and hydroxycellulose, with a rate-limiting membrane and an adhesive layer on the skin side. The “matrix design” (or “drug-in-adhesive”) formulation, on the other hand, consists of an adhesive layer of solid silicone in which the substance is suspended [23]. The two formulations have a similar substance release pattern and are both widely used for the management of chronic pain in out-of-hospital settings.

These devices are certainly suitable for abusive consumption, which can occur either by transdermal absorption or through other atypical and sometimes notably inventive routes of administration [24,25]. Atypical methods of consumption described in the literature involve the application of the patch on the buccal mucosa, ingestion, or chewing it, combining mechanical and thermal effects in increasing the release of the substance with a notably higher rate of absorption [26,27,28,29,30]. Following the massive release of the substance from the patch, in fact, this is rapidly absorbed through biological barriers constituted by the digestive tract mucosa, which is intrinsically much more permeable and vascularized than the superficial layers of the skin. Moreover, a portion of the substance is absorbed through the oral mucosa and is not affected by the first pass effect through the portal circulation, typical in the case of gastrointestinal absorption, resulting in even higher bioavailability.

Similar mechanisms intervene in the case of transrectal absorption, also described in the case of death from a fentanyl overdose [31]. Another group of atypical routes of administration, on the other hand, is characterized by the extraction of the substance from the patch and its subsequent consumption. In the case of a “reservoir design” patch, it is easily accessible and extractable using a needle [32]. In the case of a “matrix design” patch, as it is not possible to directly aspirate the compound, its extraction is carried out with other methods, such as simmering in hot water [33]; other substances (such as citric acid added to sterile water, methanol, ethanol, dichloromethane, and hot acetone) are also reported to be used as solvents [34]. The substance thus obtained is commonly directly injected intravenously, drunk, or inhaled by volatilization [35,36,37,38]. Finally, some cases of “vaporization”, performed by cutting a frozen patch into pieces, placing it in an aluminum foil, and heating it, are reported in the scientific literature [34,35] (Table 3).

In all the listed cases, however, the intake of large quantities of fentanyl (between 2.5 and 10 mg per dose), capable of guaranteeing therapeutic dosages for around 72 h with the proper administration route, occurs in a rapid and uncontrolled manner. In particular, the application of fentanyl patches on broken skin is able to provide 5-fold faster absorption, increased to approximately 30-fold through tissues without a stratum corneum (such as oral or respiratory mucosa) [39,40,41]. These absorption pathways guarantee the achievement of high blood concentrations in short time intervals and allow the achievement of the psychotropic and/or analgesic effects sought by consumers/abusers; on the other hand, associated with the drug′s narrow therapeutic window, it exposes them to a high risk of acute toxicity and death from overdose [42,43].

Regarding the post-mortem blood concentration, a retrospective study conducted on fentanyl-related deaths in the province of Ontario showed that, in cases in which fentanyl was recognized as the sole cause of death (*n* = 54), the blood concentration of this substance showed considerable variability, ranging from 3 to 383 ng/mL, with an average value of 25 ng/mL. Furthermore, a partial overlap was found with the blood values relating to cases of death due to natural causes, in which the finding of fentanyl was considered accidental (*n* = 12, range: 2.7–33 ng/mL, mean 12 ng/mL) [44].

Thompson et al. analyzed a 23-case series, divided into three groups based on the role of fentanyl in the determination of death [45]. In the group of fentanyl-only overdose and mixed-drug overdose, the blood concentration of fentanyl ranged from 5 to 120 ng/mL (*n* = 8, mean 36 ng/mL) and from 5 to 152 ng/mL (*n* = 11, mean 31 ng/mL), respectively. Values from the group with the incidental finding of fentanyl (in which death occurred due to natural causes and fentanyl was mainly administered for chronic pain therapy) showed some overlap in concentrations with the other two groups (*n* = 4, range 2–15 ng/mL, mean 5 ng/mL). In this regard, evidence from clinical practice suggests that effective postoperative analgesia is guaranteed for lower serum concentrations in opioid-naive subjects (0.63–1.5 ng/mL); suppressive effects on respiratory function, in the same category, are already observable from concentrations higher than 1.5 ng/mL, while deeper sedation, apnea, and loss of protective airway reflexes occur at concentrations higher than 3 ng/mL [41].

The presence of such considerable variability in concentration, as well as the finding of high values of blood fentanyl even in subjects consuming fentanyl who die from other causes, constitute two major issues in the interpretation of fentanyl-related deaths, resulting in the difficulty of identifying a quantitative cut-off above which fentanyl’s contribution in the determination of death can be considered relevant.

This aspect can be justified by the presence of tolerance phenomena in habitual consumers, but also by the presence of demonstrated post-mortem redistribution phenomena already consistent in the first few hours following death, difficult to predict and quantify [46,47].

For this reason, in order to confirm the effective role of exogenous intoxication in the determination of death and exclude the existence of further competing elements, the finding of a high concentration of blood fentanyl should not be considered alone, but must necessarily be contextualized with the circumstantial, pharmacological, toxicological, and forensic pathological elements available [46].

The high mortality rate among abusive users, on the other hand, cannot be justified solely by the high dosage used and has led to the deepening of the most intimate pharmacokinetic and pharmacodynamic mechanisms [48]. First of all, it was noticed that respiratory depression induced by fentanyl not only affects the respiratory rate, but also the tidal volume [48]. This effect is thought to be a consequence of an increase in thoracic muscular stiffness, resulting in an important obstacle to the physiological respiratory mechanics [49,50,51]. Fentanyl-induced lethal respiratory failure generally occurs significantly faster than with classic opioids for abuse, appearing in approximately two minutes after intravenous injection [52]. Moreover, the effects of fentanyl are less easily antagonized by the administration of naloxone [53,54], while maintaining good sensitivity to the effects of diprenorphine. These last two elements clearly pose objective difficulties in rescuing overdosed subjects, as the intervention of health professionals must be very timely and requires rapid recognition of the consumed substances. In view of its highly intrinsic effect, fentanyl also appears to be able to partially bypass the tolerance mechanisms induced by abused opioids [48,55], easily causing respiratory failure even in regular users. Finally, it should be remembered that the consumption of fentanyl is generally associated with the intake of other psychotropic substances, which can enhance or play a synergistic role in its deleterious effects.

According to a recent systematic review conducted on fentanyl-related deaths, in fact, simultaneous drug use was commonly reported; in detail, other opiates (37% of the total deaths), antidepressant/antipsychotic drugs (17%), cocaine (15%), and benzodiazepines (14%) were the most frequently associated substances in the case of fatality [56].

In the present case, findings collected during post-mortem investigations allowed us to identify a decisive role of fentanyl consumption in the cause of death. In fact, the blood concentration of fentanyl was, consistently with the scientific literature, adequate to trigger lethal respiratory suppression. This finding is, moreover, consistent with the set of toxicological and circumstantial data, and with the evidence of forensic pathological nature, which reflects the more classic alterations related to cases of intoxication by opioids or opioid receptor agonists.

In contrast, the concentration of ethyl alcohol was low and not associated with death; at the same time, the positivity for BEG in blood and urine, and cocaine in urine, was suggestive of previous cocaine consumption without any toxicological effects involved in the cause of death. The same can be said about morphine and 6-monoacethylmorphine: the presence of free morphine and 6-monoacethylmorphine only in urine can be attributed to the previous consumption of heroin, while the presence of conjugated morphine (positive total morphine) in the blood may be due to post-mortem redistribution from enterohepatic circulation. The intake of fentanyl presumably took place via the chewing of a transdermal patch with the protective film still attached; following the aspiration of the protective film in the left main bronchus, the transdermal patch was probably eliminated by vomiting. The pathophysiology of death can be identified as combined respiratory failure, in which both central suppression and a fentanyl-induced increase in muscular stiffness played a substantial role; a further contribution, albeit minor, in determining a mechanical obstruction to the flow of air in the airways could have been caused by the presence of the protective film at the bronchial level.

## 6. Conclusions

According to a comprehensive diagnosis based on the autopsy findings and toxicological analyses, the cause of death has been identified as fentanyl intoxication. This is an unusual autopsy case of intoxication capable of shedding light on some worrying aspects of drug abuse in the present era. In fact, the abusive use of fentanyl is a considerably important issue, burdened by a high rate of complications and lethality for the aforementioned pharmacological reasons [4]; the assumption of this substance through an atypical route of consumption is, on the other hand, a rare but increasing occurrence, which can remain unrecognized in the course of clinical evaluation or post-mortem investigation.

From this point of view, in fact, the role of pathology and forensic toxicology must not end at the collection and documentation of scientific evidence for justice purposes, but should also guarantee the provision of a window on some underestimated social and health problems in order to address the necessary social and health policies [57,58,59,60,61,62,63,64,65].

## Figures and Tables

**Table 1:** LODs and LOQs for all substances analyzed in the study.

Compound	LOD	LOQ
Methadone	5 ng/mL	15 ng/mL
EDDP	5 ng/mL	15 ng/mL
Morphine	5 ng/mL	15 ng/mL
6-MAM	5 ng/mL	15 ng/mL
Fentanyl	1.5 ng/mL	2.5 ng/mL
Alprazolam	3 ng/mL	10 ng/mL
Cocaine	5 ng/mL	15 ng/mL
BEG	10 ng/mL	30 ng/mL
Ethyl alcohol	0.05 g/L	0.1 g/L

**Table 2:** Results of toxicological analysis.

Compound	Femoral Blood	Urine
Methadone	<LOD	195 ng/mL
EDDP	<LOD	285 ng/mL
Total morphine	133 ng/mL	5553 ng/mL
Free morphine	<LOD	995 ng/mL
6-MAM	<LOD	182 ng/mL
Fentanyl	50 ng/mL	73 ng/mL
Alprazolam	<LOD	96 ng/mL
Cocaine	<LOD	994 ng/mL
BEG	91 ng/mL	4967 ng/mL
Ethyl alcohol	0.22 g/l	-

**Table 3:** Different methods of extraction/consumption of fentanyl contained in an adhesive patch.

Extraction/Consumption Method	Characteristics
Application of a patch on cutaneous surface	- Release of the substance occurs in a controlled manner, guaranteeing approximately constant absorption for a few days; - Heating or applying a patch on broken skin slightly increases the absorption rate.
Chewing or ingestion of a patch—Application of a patch to buccal or rectal mucosa	- Release of the substance from the patch is slightly increased due to the combination of mechanical and thermal effects; - Substance is rapidly absorbed through digestive tract mucosa, which is intrinsically much more permeable and vascularized than the skin; - A variable portion of substance is absorbed through oral or rectal mucosa and is not affected by first pass effect.
Needle extraction of fentanyl from a patch	- Only viable for “reservoir design” patches; - Pharmacokinetics strictly depends on the following consumption method.
Extraction by simmering in hot water	- Also viable for “matrix design” patches; - Release of substance from the patch is mainly determined by thermal effect; - Pharmacokinetics strictly depends on the following consumption method.
Extraction using other solvents	- Also viable for “matrix design” patches; - Citric acid added to sterile water, methanol, ethanol, dichloromethane, and hot acetone are frequently used; - Pharmacokinetics strictly depends on the following consumption method.
Patch smoking or “vaporization”	- Performed by cutting a frozen patch into pieces, heating it, and inhaling the vapors; - Effects of inhaled vapors appear rapidly; on the other hand, only a portion of the substance is absorbed.

## Data Availability

The data used to support the findings of this study are available from the corresponding author upon request.
